# Fibromuscular Dysplasia and Spontaneous Cervical Artery Dissection

**DOI:** 10.1001/jamanetworkopen.2025.40800

**Published:** 2025-11-06

**Authors:** Ahmad Nehme, Liqi Shu, Marion Boulanger, Junyue Ma, Salter Arms, Daniel Mandel, Christopher R. Leon Guerrero, Esther S. H. Kim, Nils Henninger, Jayachandra Muppa, Mirjam R. Heldner, Kateryna Antonenko, Valentin Steinsiepe, Marcel Arnold, Setareh Salehi Omran, Ross Crandall, Evan Lester, Aaron Rothstein, Ossama Khazaal, Malik Ghannam, Mohammad Almajali, Edgar A. Samaniego, Bastien Rioux, Alexandre Y. Poppe, Ana Catarina Fonseca, Michele Romoli, Marialuisa Zedde, David S. Liebeskind, Brian Mac Grory, Wayneho Kam, Sami Al Kasab, Mary Penckofer, Adeel Zubair, Richa Sharma, João Pedro Marto, Balaji Krishnaiah, Cheran Elangovan, Marwa Elnazeir, Farhan Khan, Shadi Yaghi, Emmanuel Touzé

**Affiliations:** 1 Neurology, Université Caen-Normandie, CHU de Caen-Normandie, Caen, France; 2 Department of Neurology, The Warren Alpert Medical School of Brown University, Brown Medical School, Providence, Rhode Island; 3 Department of Neurology, Atrium Health, Charlotte, North Carolina; 4 Sanger Heart and Vascular Institute, Atrium Health, Charlotte, North Carolina; 5 Department of Neurology, University of Massachusetts Chan Medical School, Worcester, Massachusetts; 6 Department of Neurology, Inselspital, University of Bern, Bern, Switzerland; 7 Department of Neurology, University of Colorado, Aurora; 8 Department of Neurology, Weill Cornell Medicine, New York, New York; 9 Department of Neurology, University of Pennsylvania, Philadelphia; 10 Department of Neurology, University of Iowa, Iowa City; 11 Department of Neurosciences, Université de Montréal, Montreal, Quebec, Canada; 12 Department of Neurology, Hospital de Santa Maria, Faculdade de Medicina da Universidade de Lisboa, Lisbon, Portugal; 13 Department of Neurosciences, Bufalini Hospital, Cesena, Italy; 14 Stroke Unit, Neurology Unit, Azienda Unità Sanitaria Locale-IRCCS di Reggio Emilia, Reggio Emilia, Italy; 15 Department of Neurology, University of California, Los Angeles; 16 Department of Neurology, Duke University, Durham, North Carolina; 17 Comprehensive Stroke Center, University of North Carolina Health Rex, Raleigh; 18 Department of Neurology and Neurosurgery, Medical University of South Carolina, Charleston; 19 Department of Neurology, Cooper University, Camden, New Jersey; 20 Department of Neurology, Yale New Haven Hospital, New Haven, Connecticut; 21 Department of Neurology, Hospital de Egas Moniz, Centro Hospitalar Lisboa Ocidental, Lisbon, Portugal; 22 Department of Neurology, University of Tennessee Health Science Center, Memphis; 23 Department of Neurology, University of Louisville, Louisville, Kentucky

## Abstract

**Importance:**

Fibromuscular dysplasia (FMD) is found in 6 to 14% of patients with spontaneous cervical artery dissection (SCEAD) and may be associated with recurrent SCEAD.

**Objective:**

To evaluate the correlates of FMD in patients with SCEAD and to determine whether FMD is associated with recurrent SCEAD.

**Design, Setting, and Participants:**

This cohort study included patients from the Stroke Prevention in Cervical Artery Dissection (STOP-CAD) retrospective cohort study who presented from January 2015 to December 2022. This multicenter and international cohort included consecutive adult patients presenting at acute care hospitals and diagnosed with SCEAD in 63 sites in 16 countries. Data were analyzed from April to November 2024.

**Exposure:**

Fibromuscular dysplasia was defined as either a history of FMD or presence of FMD on cervical or renal artery imaging.

**Main Outcomes and Measures:**

Clinical and radiological correlates were compared between patients with and without FMD using logistic regression models. Rates of recurrent SCEAD by 24 months were compared using a Cox proportional hazards model.

**Results:**

This study included 3714 patients with SCEAD (median [IQR] age, 47 [38-56] years; 1637 [44.1%] females), of whom 196 (5.3%) had FMD. Patients with FMD were older (aOR per 10 years, 1.28; 95% CI, 1.14-1.43) and more often female (aOR, 2.00; 95% CI, 1.45-2.75). They more often had a history of dissection involving a noncervical artery (aOR, 8.10; 95% CI, 2.64-24.83), a history of SCEAD (aOR, 2.05; 95% CI, 1.07-3.93), a recent upper respiratory tract infection (aOR, 2.40; 95% CI, 1.52-3.78), a cerebral aneurysm (aOR, 2.22; 95% CI, 1.22-4.06), or a history of migraines (aOR, 2.44; 95% CI, 1.75-3.40). On imaging, they were less likely to have a single vertebral artery dissection (aOR, 0.37; 95% CI, 0.25-0.55) or an occlusive dissection (aOR, 0.55; 95% CI, 0.38-0.78). Eighty-one patients experienced a recurrent SCEAD, of which 46 (56.8%) occurred in the first 3 months of follow-up. The 24-month risk of recurrent SCEAD was 7.7% (95% CI, 3.1%-12.2%) and 2.8% (95% CI, 2.1%-3.5%) in patients with and without FMD, respectively (aHR, 2.75; 95% CI, 1.46-5.18; *P* = .002).

**Conclusions and relevance:**

In this cohort study of patients with SCEAD, FMD was associated with distinct correlates and a higher rate of recurrent SCEAD. These findings may help physicians in identifying and counseling patients with FMD and SCEAD.

## Key Points

**Question:**

What are the correlates of fibromuscular dysplasia (FMD) in patients with spontaneous cervical artery dissection (SCEAD), and is FMD associated with recurrent SCEAD?

**Findings:**

In this cohort study of 3714 patients with SCEAD, the presence of FMD was associated with distinct clinical and radiological correlates, as well as a 2.75-fold higher rate of recurrent SCEAD.

**Meaning:**

These findings suggest that patients with FMD may represent a separate subgroup within the SCEAD population, with distinct correlates and a higher rate of recurrent SCEAD.

## Teaser

This cohort study evaluates fibromuscluar dysplasia (FMD) in patients with spontaneous cervical artery dissection (SCEAD) to determine if FMD is associated with SCEAD using an international cohort.

## Introduction

Fibromuscular dysplasia (FMD) is an idiopathic, systemic, noninflammatory and nonatherosclerotic disease of small to medium-sized arteries that most often manifests as beaded multifocal lesions in renal, extracranial carotid, and extracranial vertebral arteries^1^ and predominantly affects females.^2,3,4,5^ FMD is a well-established risk factor for arterial dissection and has been associated with arterial stenosis, aneurysms, and tortuosity.^1^

Cervical FMD is found in 6 to 14% of patients with spontaneous cervical artery dissection (SCEAD).^6,7,8,9,10^ Up to 40% of patients with SCEAD evaluated with whole-body computed tomography angiography (CTA) have at least 1 arterial bed with FMD, albeit no data are available in consecutively selected patients.^3,11^ Hypertension and migraine are risk factors for SCEAD and are more prevalent in patients with FMD.^12,13,14,15^ Among patients with FMD, males are more likely than females to present with arterial dissection.^16,17^

Previous studies evaluating the association between cervical FMD and SCEAD found that patients with FMD may have a higher rate of recurrent SCEAD.^6,18^ However, it is unknown whether these results are generalizable to other populations, and further data are needed to counsel patients with FMD and SCEAD. Therefore, using data from a large, international, multicenter cohort study of patients with SCEAD, we evaluated the clinical and radiological correlates of FMD in patients with SCEAD and whether FMD is associated with recurrent SCEAD.

## Methods

This cohort study is reported in accordance with the Strengthening the Reporting of Observational Studies in Epidemiology (STROBE) reporting guideline.^19^ This analysis was conducted in accordance with the ethical guidelines of the original Antithrombotic Treatment for Stroke Prevention in Cervical Artery Dissection (STOP-CAD) study, which received approval at Lifespan, without a requirement for informed consent. Ethical approval was obtained at other sites as per local regulations. For this secondary analysis of deidentified data, the Lifespan institutional review board waived the requirement for additional approval.

### Study Design and Patient Population

We performed a secondary analysis of the STOP-CAD study, whose methods have been previously reported.^20^ Briefly, STOP-CAD was an international, retrospective, observational cohort study that included consecutive hospitalized patients with cervical or intracranial artery dissection unrelated to a major trauma at 63 sites from 16 countries from January 2015 to December 2022. Patients were identified using administrative coding or local registries, and local investigators confirmed the diagnosis of dissection after review of medical records and imaging. Iatrogenic, incidental, and chronic dissections were excluded. Due to differences in pathophysiology between cervical and intracranial artery dissection,^21^ this substudy was restricted to patients with SCEAD with or without intracranial extension, while patients with isolated intracranial dissection were excluded.

### Study Variables

Fibromuscular dysplasia was defined as either a history of FMD or the presence of FMD affecting cervical or renal arteries, diagnosed with any vascular imaging modality, as requested by the treating physician (duplex ultrasonography, CTA, magnetic resonance angiography, or digital subtraction angiogram). History of FMD was assessed by reviewing medical records. The presence of FMD was evaluated by local investigators who had access to source images and radiology reports while unblinded to patient features and follow-up data. Local investigators did not further subtype FMD as focal or multifocal. Local investigators were asked to report if they considered that FMD was exclusively present on a dissected arterial segment. Since acute or chronic dissection-related changes can mimic FMD and lead to misdiagnosis of the disease, patients were excluded from the primary analysis if investigators considered that FMD was exclusively present on a dissected arterial segment.^1,22^ Images were not centrally reviewed. No systematic imaging of extracervical arteries was mandated, but patients with SCEAD in whom renal FMD was found on any imaging were included in the FMD group, as per the FMD consensus criteria.^1^

Variables were extracted from the medical records and by review of source images by local investigators. Queried variables included demographics (age, sex), prior comorbidities (hypertension, dyslipidemia, diabetes, migraine, SCEAD, dissection involving a noncervical artery, aortic aneurysm, cerebral aneurysm), habits (active smoking), dissection triggers (recent infection, minor trauma, postpartum period), clinical presentation (ischemic stroke, transient ischemic attack, headache, cervical pain, tinnitus, cranial nerve palsy), and imaging features (acute dissection site[s], arterial occlusion [no residual flow], dissecting aneurysm, partially occlusive thrombus, cerebral aneurysm).

### Outcomes

Recurrent SCEAD was defined as occurring at least 7 days after diagnosis of the initial SCEAD and had to affect a different artery or a different site of the same artery. This definition was chosen to avoid classifying dynamic changes at the initial dissection sites as recurrent events. Local investigators identified recurrent SCEAD. The primary investigator (S.Y.) of STOP-CAD reviewed imaging reports at the time of recurrence to ensure that the events met the study definition.

### Statistical Analysis

Baseline patient characteristics, SCEAD presentation, and imaging features in patients with and without FMD were compared using logistic regression models. A mixed-effects logistic regression model with a random intercept for sites was used in multivariable analyses to account for within-group clustering. The absolute risks of recurrent SCEAD were calculated using Kaplan-Meier survival estimates at 3 and 24 months and compared between the FMD and no-FMD groups with the log-rank test and a Cox proportional hazards model with shared frailty, to account for within-group dependence at the site level. Patients entered the cohort at the date of SCEAD diagnosis (index date) and were followed until the first of recurrent SCEAD, death, loss to follow-up, or 24 months. Recurrence rates were also compared in a multivariable model including variables that were found to be associated with recurrent SCEAD in a recent systematic review (age [continuous] and migraine).^6,23,24,25^ The proportional hazards assumption was verified by inspecting survival curves and using Schoenfeld residual tests. To determine if the association between FMD and SCEAD varied according to sex, the presence of multivessel SCEAD at the time of diagnosis, or time (less than 3 months vs 3 to 24 months), the corresponding interaction terms were added to the Cox models.

In STOP-CAD, the development of an ischemic stroke was captured during the first 180 days of follow-up.^20^ The absolute risks of ischemic stroke were calculated using Kaplan-Meier survival estimates at 180 days and compared between the FMD and no-FMD groups with the log-rank test and a Cox proportional hazards model with shared frailty. Patients entered the cohort at the date of SCEAD diagnosis (index date) and were followed until the first of ischemic stroke, death, loss to follow-up, or 180 days.

In sensitivity analyses, models for SCEAD recurrence were repeated (1) restricting the definition of FMD to cervical FMD, (2) including patients in the no-FMD group when investigators considered that FMD was exclusively present on a dissected arterial segment, (3) excluding patients with a history of SCEAD, (4) limiting the population to patients with at least 1 follow-up imaging of cervical arteries, and (5) accounting for the competing risk of death (Fine-Gray model). Due to their low number (less than 1%), missing data were not imputed. A 2-sided *P* < .05 was considered statistically significant. Analyses were performed with Stata version 18 (StataCorp). Data were analyzed from April to November 2024.

## Results

### Baseline Demographics and Characteristics

Among the 4023 patients included in STOP-CAD, 3714 were eligible for this study (median [IQR] age, 47 [38-56] years; 1637 females [44.1%]). Reasons for exclusion were isolated intracranial dissection (182 [4.5%]), investigators considering that FMD was exclusively present on a dissected arterial segment (122 [3.0%]), and no documentation of FMD status (5 [0.1%]).

A history of FMD prior to SCEAD diagnosis was reported in 15 (0.4%) patients, who all had FMD on the cervical or renal artery imaging obtained before or at the time of the SCEAD. In total, FMD was detected in 196 patients (5.3%) and involved the cervical arteries in 183 (4.9%). Imaging of the renal arteries was performed in 433 of all included patients (11.7%), of whom 39 (9.0%) had renal FMD. Imaging of the renal arteries was obtained in 75 (41.0%) and 358 (10.1%) of patients with and without cervical FMD, respectively. Of the 39 patients with renal FMD, 26 also had cervical FMD.

### Clinical and Radiological Correlates of FMD in Patients With SCEAD

Patients with FMD were more often female than patients without FMD (Table 1). Prior comorbidities differed between the 2 groups, as patients with FMD had a higher likelihood of migraine headache, prior SCEAD or prior dissection involving noncervical arteries, and a lower likelihood of diabetes or active smoking. A recent upper respiratory tract infection was twice as frequent in patients with FMD, with no difference in other dissection triggers. After restricting the population to patients with an ischemic stroke at presentation, FMD remained associated with a recent upper respiratory tract infection. The prevalence of cerebral aneurysms (known or discovered on vascular imaging) was 3-fold higher in patients with vs without FMD. These patient features and older age were significantly associated with FMD in the multivariable model, except for active smoking. Patients with FMD were more often included from North American sites, where the proportion undergoing renal artery imaging (153 [6.8%]) was lower than other regions (280 [19.3%]).

**Table 1.** Baseline Features and Dissection Triggers in Patients With Spontaneous Cervical Artery Dissection With and Without Fibromuscular Dysplasia

Characteristic	FMD (n = 196)	No FMD (n = 3518)	OR (95% CI)	
			Univariable	Multivariable^a^
Median (IQR) age, y	49 (40-56)	46 (37-56)	1.10 (0.99-1.22)^b^	1.28 (1.14-1.43)^b^
Sex				
Male	74 (37.8)	2003 (56.9)	1 [Reference]	1 [Reference]
Female	122 (62.2)	1515 (43.1)	2.18 (1.62-2.93)	2.00 (1.45-2.75)
Country of inclusion				
North America	142 (72.5)	2120 (60.3)	1 [Reference]	NA
South America	2 (1.0)	138 (3.9)	0.16 (0.05-0.88)	NA
Europe/Middle East	51 (26.0)	1211 (34.4)	0.63 (0.45-0.87)	NA
Other	1 (0.5)	49 (1.4)	0.30 (0.04-2.22)	NA
Arterial hypertension	69 (35.2)	1228 (34.9)	1.01 (0.75-1.37)	NA
Dyslipidemia	45 (23.0)	807 (22.9)	1.00 (0.71-1.41)	NA
Diabetes	9 (4.6)	310 (8.8)	0.50 (0.25-0.98)	0.46 (0.23-0.92)
Active smoking	25 (12.8)	693 (19.7)	0.60 (0.39-0.91)	0.69 (0.45-1.06)
Migraine headache	68 (34.7)	550 (15.6)	2.87 (2.11-3.90)	2.44 (1.75-3.40)
With aura	19 (9.7)	176 (5.0)	NA	NA
Without aura	30 (15.3)	262 (7.4)	NA	NA
Migraine type unknown	19 (9.7)	112 (3.2)	NA	NA
Prior cervical artery dissection	13 (6.6)	80 (2.3)	3.05 (1.67-5.59)	2.05 (1.07-3.93)
Prior dissection of a noncervical artery	5 (2.6)	12 (0.3)	7.65 (2.67-21.93)	8.10 (2.64-24.83)
Cerebral aneurysm (known or discovered)	15 (7.7)	89 (2.5)	3.19 (1.81-5.63)	2.22 (1.22-4.06)
Aortic aneurysm	1 (0.5)	18 (0.5)	1.00 (0.13-7.51)	NA
Recent upper respiratory tract infection	25 (12.8)	210 (6.0)	2.30 (1.48-3.58)	2.40 (1.52-3.78)
Recent minor trauma^a^	47 (24.0)	792 (22.5)	1.09 (0.77-1.52)	NA
Postpartum period	4/122 (3.3)	46/1515 (3.0)	1.08 (0.38-3.06)	NA
Pregnancy	0/122 (0)	7/1515 (0.5)	0.82 (0.05-14.46)	NA

Abbreviations: FMD, fibromuscular dysplasia; NA, not applicable; OR, odds ratio.

^a^ Missing in 1 patient.

^a^ Model includes study site, age (continuous), female sex, diabetes, active smoking, migraine headache, prior cervical artery dissection of a noncervical artery, cerebral aneurysm (known or discovered), and recent upper respiratory tract infection.

^b^ OR per 10 years.

Patients with FMD less often had an ischemic stroke or a transient ischemic attack at SCEAD presentation (131 [66.8%] vs 2731 [77.6%]; odds ratio, 0.58; 95% CI, 0.43-0.79; *P* = .001). In univariable analyses, factors associated with both FMD and ischemic symptoms at presentation included older age, male sex, diabetes, active smoking, as well as absence of a recent upper respiratory tract infection, a previous SCEAD, or a previous dissection involving a noncervical artery. In the multivariable model including these variables, FMD was no longer significantly associated with a lower risk of ischemic stroke or transient ischemic attack at SCEAD presentation (aOR, 0.80; 95% CI, 0.58-1.10; *P* = .17).

Patients with FMD more frequently presented with headache or cervical pain (Table 2). Dissection-related imaging findings differed, as patients with FMD had a lower prevalence of occlusive dissections and a higher prevalence of dissecting aneurysms. Dissection sites also varied between groups: patients with FMD had more multiple dissections and less single vertebral artery dissections. In the multivariable model, patients with FMD had a significantly lower likelihood of presenting with a single vertebral artery dissection or an occlusive dissection.

**Table 2.** Presenting Clinical and Radiological Features in Patients With Spontaneous Cervical Artery Dissection With and Without Fibromuscular Dysplasia

Characteristic	Patients, No. (%)		OR (95% CI)	
	FMD (n = 196)	No FMD (n = 3518)		
			Univariable	Multivariable^a^
Headache	112 (57.1)	1680 (47.8)	1.46 (1.09-1.95)	1.15 (0.84-1.57)
Cervical pain	75 (38.3)	1063 (30.2)	1.43 (1.06-1.93)	1.56 (1.12-2.17)
Tinnitus	7 (3.6)	95 (2.7)	1.33 (0.61-2.92)	NA
Cranial nerve palsy	1 (0.5)	46 (1.3)	0.39 (0.05-2.82)	NA
Acute cervical artery dissection site				
Single carotid	116 (59.2)	1812 (51.5)	1 [Reference]	NA
Single vertebral	40 (20.4)	1370 (38.9)	0.46 (0.32-0.66)	0.37 (0.25-0.55)
Multiple	40 (20.4)	336 (9.6)	1.86 (1.27-2.71)	1.44 (0.95-2.16)
Dissecting aneurysm	39 (19.9)	445 (12.6)	1.72 (1.19-2.47)	1.28 (0.86-1.89)
Partially occlusive thrombus	12 (6.1)	194 (5.5)	1.12 (0.61-2.04)	NA
Occlusive dissection^b^	48 (24.5)	1314 (37.8)	0.53 (0.38-0.75)	0.55 (0.38-0.78)

Abbreviations: FMD, fibromuscular dysplasia; NA, not applicable; OR, odds ratio.

^a^ Model includes study site, headache, cervical pain, acute cervical artery dissection site, and occlusive dissection.

^b^ Missing in 38 patients.

### Association Between FMD and Recurrent SCEAD

The median (IQR) follow-up was 211 (98-651) days and 300 (98-720) days in patients with and without FMD, respectively. Baseline characteristics were similar between patients with and without complete follow-up (eTable 1 in Supplement 1). A total of 91 patients died prior to reaching 24 months of follow-up or developing a recurrent SCEAD, representing 2 patients with FMD (1.0%) and 89 patients without FMD (2.5%). The median (IQR) number of repeat imaging of cervical arteries in the 24-month follow-up period was 1 (1-2) in patients with FMD and 1 (0-2) in patients without FMD.

**Figure 1.**
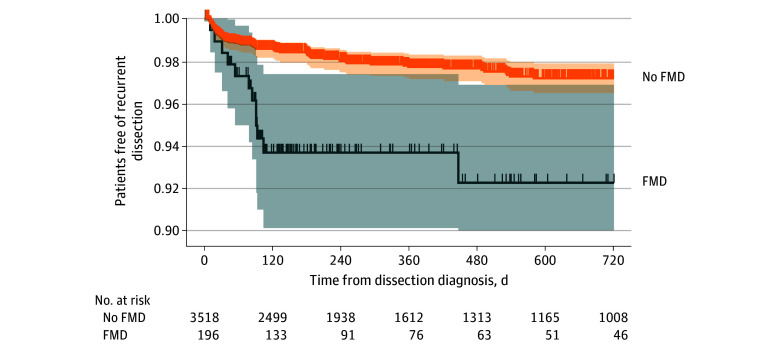
Time to Recurrent Spontaneous Cervical Artery Dissection (SCEAD) in Patients With SCEAD, Stratified According to the Presence of Fibromuscular Dysplasia (FMD) Survival curves obtained with the Kaplan-Meier formula. Shaded areas represent 95% CIs.

Eighty-one patients (2.2%) experienced a recurrent SCEAD, of whom 12 (14.8%) had FMD. Forty-six (56.8%) recurrent SCEAD occurred in the first 3 months of follow-up. At 3 months, the risk of recurrent SCEAD was 3.9% (95% CI, 1.0%-6.6%) in patients with FMD and 1.4% (95% CI, 0.9%-1.8%) in patients without FMD (*P* < .001). Corresponding 24-month risks were 7.7% (95% CI, 3.1%-12.2%) and 2.8% (95% CI, 2.1%-3.5%) (*P* < .001) (Figure 1). The median (IQR) time to recurrent SCEAD was 53 (18-180) days. FMD was significantly associated with recurrent SCEAD in univariable (hazard ratio [HR], 2.93; 95% CI, 1.57-5.47; *P* = .001) and multivariable analyses (adjusted hazard ratio [aHR], 2.75; 95% CI, 1.46-5.18; *P* = .002). The higher rate of recurrent SCEAD in patients with FMD was present in all sensitivity analyses (Table 3). There was no significant interaction with sex, the presence of multivessel SCEAD at the time of diagnosis, or time (less than 3 months vs 3 to 24 months). The site of recurrent SCEAD according to FMD status is reported in eTable 2 in Supplement 1.

**Table 3.** Association Between Fibromuscular Dysplasia and the Rate of Recurrent Spontaneous Cervical Artery Dissection (Sensitivity Analyses)

Analysis	Sample size	Median (IQR) follow-up, d	No. of recurrent SCEAD	Model 1		Model 2	
				HR (95% CI)^a^	*P* value	HR (95% CI)^b^	*P* value
Defining FMD only as FMD on a nondissected cervical arterial segment	3714	296 (98-720)	81	2.93 (1.57-5.47)	.001	2.74 (1.45-5.17)	.002
Including patients with FMD exclusively reported on a dissected arterial segment in the no-FMD group	3836	295 (97-720)	88	2.93 (1.75-4.90)	<.001	2.71 (1.61-4.56)	<.001
Excluding patients with a history of SCEAD	3621	295 (97-720)	74	3.12 (1.63-5.98)	.001	2.88 (1.48-5.59)	.002
Restricting the population to patients with at least 1 follow-up image of the cervical arteries	2764	360 (145-720)	81	2.86 (1.53-5.32)	.001	2.68 (1.42-5.06)	.002
Accounting for the competing risk of death	3714	296 (98-720)	81	3.12 (1.88-5.17)	<.001	2.81 (1.68-4.72)	<.001

Abbreviations: FMD, fibromuscular dysplasia; HR, hazard ratio; SCEAD, spontaneous cervical artery dissection.

^a^ Model 1 includes study site.

^b^ Model 2 includes study site, age (continuous) and history of migraine headache.

### FMD and Ischemic Stroke During Follow-Up

**Figure 2.**
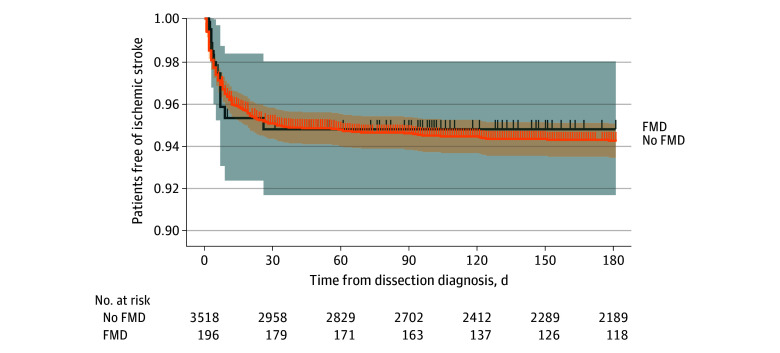
Time to Ischemic Stroke in Patients With Spontaneous Cervical Artery Dissection, Stratified According to the Presence of Fibromuscular Dysplasia (FMD) Survival curves obtained with the Kaplan-Meier formula. Shaded areas represent 95% CIs.

During the first 180 days of follow-up, an ischemic stroke occurred in 10 patients with FMD (5.1%) and 190 (5.4%) patients without FMD (Figure 2). The 180-day risk of ischemic stroke was 5.2% (95% CI, 2.0%-8.3%) in patients with FMD and 5.8% (95% CI, 5.0%-6.6%) in patients without FMD (*P* = .78) (Figure 2). Fibromuscular dysplasia was not associated with the 180-day risk of ischemic stroke (HR, 0.85; 95% CI, 0.45-1.63; *P* = .64).

## Discussion

In this large multicenter cohort study of patients with SCEAD, we found that FMD was associated with differences in clinical and radiological features as well as a higher rate of SCEAD recurrence. Consistent with prior studies, patients with FMD were more often female, had a higher prevalence of cerebral aneurysms, and more frequently had a history of migraine headache.^6^ In addition, they were more likely to report a recent upper respiratory tract infection. In the first 24 months after diagnosis, the rate of recurrent SCEAD was nearly 3-fold higher in patients with FMD. Most recurrent SCEADs occurred in the first 3 months of follow-up, irrespective of the presence of FMD.

The prevalence of FMD reported in our population of patients with SCEAD (5%) is consistent with recent reports (6% to 8%)^6,7^ but lower than in older studies (13% to 14%).^8,9^ Potential reasons for this discrepancy include more frequent use of digital subtraction angiography, possible selection biases, or misdiagnosis of dissection-related changes as FMD in previous studies.^8,9^ Our results suggest that physicians frequently attribute dissection-related changes to FMD, potentially overestimating the prevalence of the disease. Current imaging criteria for FMD require the presence of focal or multifocal arterial lesions outside of a dissected arterial segment. However, SCEAD usually occurs at the same arterial site as FMD lesions,^26^ and a previously undetected chronic dissection may mimic the appearance of FMD. Future blood-based biomarkers may overcome the limitations of these criteria but are not yet validated for clinical use.^27^ While renal FMD was part of our study definition, only 12% of patients underwent dedicated renal artery imaging, and the latter was more frequently positive in the presence of cervical FMD. Some patients may only have undergone Doppler ultrasonography, which may have a lower sensitivity for FMD than CTA.^28^ Minor forms of cervical or renal FMD may also have been missed by nonexpert raters, and other sites (eg, visceral, iliac) were not evaluated. Consequently, our studied population mostly encompassed cervical FMD, and the true prevalence of FMD in patients with SCEAD is likely to be higher.

The proportion of females with FMD and SCEAD was lower than females in the overall FMD population,^2,4,5^ consistent with previous studies suggesting that male sex is a risk factor for SCEAD in patients with FMD.^16^ The prevalence of cerebral aneurysms in our study is in agreement with prior meta-analyses performed in patients with FMD^29^ and in the general population.^30^ Investigators from North American sites more often diagnosed FMD, but it is unknown whether this reflects differences in the prevalence of FMD, FMD-specific dissection triggers, or regional diagnostic practices. A recent upper respiratory tract infection was present in a small proportion of patients but twice more frequently in the FMD group, suggesting that dissection in patients with FMD may require an external factor (eg, systemic trigger) in addition to susceptible arteries. Patients with an ischemic stroke may have been less likely to report recent exposures due to their neurological impairment, but the association between FMD and recent upper respiratory tract infection was also found after stratifying patients by SCEAD presenting mode.

As in previous hospital-based studies, most patients with SCEAD presented with an ischemic stroke or a transient ischemic attack.^12,20,31^ However, patients with FMD were less likely to present with ischemic symptoms. They had less occlusive dissections, which more often present with ischemic symptoms.^32,33,34^ Conversely, they had more multiple dissections, which may be less likely to present with ischemic symptoms.^7^ Multiple SCEADs have been previously linked to FMD and dissecting aneurysms,^7^ leading to the hypothesis that these patients may preferentially develop subadventitial rather than subintimal dissections. However, the association between FMD and a nonischemic SCEAD presentation was no longer significant after accounting for baseline patient features. One potential explanation is that increased awareness of dissection symptoms may have contributed to detection of more nonischemic SCEAD in patients with a history of previous cervical or extracranial dissection, who were more likely to have FMD.

Consistent with a multicenter Italian study, the rate of recurrent SCEAD in the first 24 months of follow-up was 3-fold higher in patients with FMD.^6^ Most recurrent dissections were identified in the first 3 months of follow-up, which may be related to temporal clustering of recurrences early after the initial diagnosis of SCEAD^6,35^ and more frequent repeat imaging during this period. After the first 5 months, very few patients with FMD developed a recurrent SCEAD, supporting that risk factors other than FMD may contribute to a transiently increased risk of recurrence. These results imply that patients and physicians should be vigilant to minor symptoms that may suggest a recurrent SCEAD during this period of increased risk, particularly in the presence of FMD.

Despite an increased risk of recurrent SCEAD, patients with FMD were not at higher risk of ischemic stroke than patients without FMD during the first 180 days of follow-up. This may be because most ischemic strokes after SCEAD are due to other mechanisms than recurrent SCEAD,^36^ with most recurrent dissections in the first year of follow-up being either asymptomatic or associated with local symptoms, as previously reported.^25^

### Limitations

This study has limitations. The STOP-CAD study was not designed to evaluate FMD; therefore, several variables were not available (eg, specific cervical arteries affected by FMD, imaging modality used to screen for renal FMD, other aneurysm sites, family history of SCEAD or FMD). Further subtyping of FMD was not performed, but most patients with FMD and SCEAD have the multifocal form.^1,2^ We cannot rule out that local investigators were more likely to identify cervical FMD or screen for renal FMD because of a recurrent SCEAD. The latter possibility is unlikely to have affected the results, as few patients were diagnosed with renal FMD, and results were unchanged after excluding these patients. The rate of recurrent SCEAD remained higher in patients with FMD after restricting the population to cases with at least 1 follow-up imaging of cervical arteries, limiting the possibility that the association resulted from an ascertainment bias. Our study provides data on the short- to medium-term risk of recurrence after SCEAD, but its results cannot be extrapolated to longer durations of follow-up.

## Conclusions

This study highlights that patients with FMD represent a distinct subgroup within the SCEAD population. The possibility of underlying FMD should be considered in patients with the identified clinical and radiological correlates. Patients with FMD may have an increased susceptibility to develop a dissection after a recent respiratory tract infection, but this requires further research. Our results validate that FMD is an important risk factor for recurrent SCEAD, with most events occurring in the first 3 months of follow-up. The results of this study mainly apply to cervical FMD, and prospective studies with systematic screening of all vascular beds, centralized imaging interpretation and longer durations of follow-up are necessary to improve our understanding of the association between FMD and SCEAD.

## Notes

Supplement 1. **eTable 1. **Baseline characteristics in patients with and without complete follow-up **eTable 2. **Recurrent spontaneous cervical artery dissection site in patients with and without fibromuscular dysplasia

Supplement 2.
Nonauthor Collaborators

Supplement 3.
Data Sharing Statement
