# Supplementary material for: Fibromuscular Dysplasia and Spontaneous Cervical Artery Dissection
Source: JAMA Netw Open. 2025 Nov 6;8(11):e2540800. doi: 10.1001/jamanetworkopen.2025.40800 (PMC12593120; doi:10.1001/jamanetworkopen.2025.40800)
Supplement: Supplement 1. — eTable 1. Baseline characteristics in patients with and without complete follow-up eTable 2. Recurrent spontaneous cervical artery dissection site in patients with and without fibromuscular dysplasia [file jamanetwopen-e2540800-s001.pdf]

# Supplemental Online Content

Nehme A, Shu L, Boulanger M, et al. Fibromuscular dysplasia and spontaneous cervical artery dissection. *JAMA Netw Open*. 2025;8(11):e2540800. doi:10.1001/jamanetworkopen.2025.40800

**eTable 1.** Baseline characteristics in patients with and without complete follow-up

**eTable 2.** Recurrent spontaneous cervical artery dissection site in patients with and without fibromuscular dysplasia

This supplemental material has been provided by the authors to give readers additional information about their work.

© 2025 Nehme A et al. *JAMA Network Open*.

**eTable 1. Baseline characteristics in patients with and without complete follow-up**

| | Recurrent dissection, death, or 24 months of follow-up (n=1224) | Loss to follow-up before recurrent dissection, death, or 24 months (n=2490) |
|---|---|---|
| Fibromuscular dysplasia (n, %) | 60 (4.9%) | 136 (5.5%) |
| Median (IQR) age (years) | 46 (37-56) | 47 (38-56) |
| Female sex (n, %) | 572 (46.7%) | 1065(42.8%) |
| Country of inclusion (n, %) | | |
| North America | 751 (61.4%) | 1511 (60.7%) |
| South America | 49 (4.0%) | 91 (3.7%) |
| Europe/Middle East | 402 (32.8%) | 860 (34.5%) |
| Other | 22 (1.8%) | 28 (1.1%) |
| Arterial hypertension (n, %) | 419 (34.2%) | 878 (35.3%) |
| Dyslipidemia (n, %) | 255 (20.8%) | 597 (24.0%) |
| Diabetes (n, %) | 112 (9.2%) | 207 (8.3%) |
| Active smoking (n, %) | 247 (20.2%) | 471 (18.9%) |
| Migraine headaches (n, %) | 206 (16.8%) | 412 (16.5%) |
| Prior cervical artery dissection (n, %) | 39 (3.2%) | 54 (2.2%) |
| Prior dissection of a non-cervical artery (n, %) | 10 (0.8%) | 7 (0.3%) |
| Cerebral aneurysm (known or discovered, n, %) | 43 (3.5%) | 61 (2.4%) |
| Recent upper respiratory infection (n, %) | 63 (5.1%) | 172 (6.9%) |

© 2025 Nehme A et al. *JAMA Network Open*.

**eTable 2. Recurrent spontaneous cervical artery dissection site in patients with and without fibromuscular dysplasia**

| Recurrent cervical artery dissection site (n, %) | No FMD (n=68) | FMD (n=12) |
|---|---|---|
| Single cervical carotid | 14 (20.6) | 6 (50.0) |
| Single cervical vertebral | 40 (58.8) | 4 (33.3) |
| Multiple | 14 (20.6) | 2 (16.7) |

One patient without FMD excluded because of unknown recurrent dissection site
FMD: fibromuscular dysplasia

© 2025 Nehme A et al. *JAMA Network Open*.
