# Supplementary material for: Fibromuscular Dysplasia and Spontaneous Cervical Artery Dissection
Source: JAMA Netw Open. 2025 Nov 6;8(11):e2540800. doi: 10.1001/jamanetworkopen.2025.40800 (PMC12593120; doi:10.1001/jamanetworkopen.2025.40800)
Supplement: Supplement 2. — Nonauthor Collaborators [file jamanetwopen-e2540800-s002.pdf]

Supplemental Online Content: Nonauthor Collaborators
*First name, last name, and suffix (if applicable) are required and will appear in PubMed.

| *Group Name(s): Stroke Prevention in Cervical Artery Dissection (STOP-CAD) investigators | | | | | | | |
|---|---|---|---|---|---|---|---|
| ***First Name and Middle Initial(s)** | ***Last Name** | ***Suffix (eg, Jr, III)** | Academic Degrees | Institution | Location (city, state/province, country) | Role or Contribution, eg, chair, principal investigator | Group (if more than 1 Group listed in the byline) and/or Subgroup (eg, Steering Committee) |
| Muhammad | Affan | | MBBS | University of Minnesota | Minnesota, Minneapolis, USA | Site investigator | |
| Omair Ul Haq | Lodhi | | MBBS | University of Minnesota | Minnesota, Minneapolis, USA | Site investigator | |
| David | Seiffge | | MD | Inselspital, University of Bern | Bern, Switzerland | Site investigator | |
| Marcel | Arnold | | MD | Inselspital, University of Bern | Bern, Switzerland | Site investigator | |
| Diego | Lopez Mena | | MD | Instituto Nacional de Neurología y Neurocirugía Manuel Velasco Suarez | Mexico City, Mexico | Site investigator | |
| Antonio | Arauz | | MD | Instituto Nacional de Neurología y | Mexico City, Mexico | Site investigator | |
| Joao Andre | Sousa | | MD | Coimbra University | Coimbra, Portugal | Site investigator | |
| Joao | Sargento-Freitas | | MD | Coimbra University | Coimbra, Portugal | Site investigator | |
| Vasco | Barata | | MD | Coimbra University | Coimbra, Portugal | Site investigator | |
| Paulo | Castro-Chaves | | MD | São João University Hospital | Porto, Portugal | Site investigator | |
| Maria Teresa | Brito | | MD | São João University Hospital | Porto, Portugal | Site investigator | |
| Muhib | Khan | | MD | Corewell Health | Grand Rapids, Michigan, USA | Site investigator | |
| Dania | Mallick | | MD | Corewell Health | Grand Rapids, Michigan, USA | Site investigator | |
| Josefin E. | Kaufmann | | Mmed | University Geriatric Medicine FELIX PLATTER, University of Basel | Basel, Switzerland | Site investigator | |
| Stefan T. | Engelter | | MD | University Geriatric Medicine FELIX PLATTER, University of Basel | Basel, Switzerland | Site investigator | |
| Christopher | Traenka | | MD | University Geriatric Medicine FELIX PLATTER, University of Basel | Basel, Switzerland | Site investigator | |
| Diana | Aguiar de Sousa | | MD | Centro Hospitalar Universitário Lisboa Central, Institute of Anatomy, Faculdade de Medicina da Universidade de Lisboa | Lisbon, Portugal | Site investigator | |
| Mafalda | Soares | | MD | Centro Hospitalar Universitário Lisboa Central | Lisbon, Portugal | Site investigator | |

Supplemental Online Content: Nonauthor Collaborators
*First name, last name, and suffix (if applicable) are required and will appear in PubMed.

| ***First Name and Middle Initial(s)** | ***Last Name** | ***Suffix (eg, Jr, III)** | Academic Degrees | Institution | Location (city, state/province, country) | Role or Contribution, eg, chair, principal investigator | Group (if more than 1 Group listed in the byline) and/or Subgroup (eg, Steering Committee) |
|---|---|---|---|---|---|---|---|
| Sara | Rosa | | MD | Centro Hospitalar Universitário Lisboa Central | Lisbon, Portugal | Site investigator | |
| Lily W | Zhou | | MD | University of British Columbia | Vancouver, Canada | Site investigator | |
| Preet | Gandhi | | MD | University of British Columbia | Vancouver, Canada | Site investigator | |
| Thalia S. | Field | | MD | University of British Columbia | Vancouver, Canada | Site investigator | |
| Steven | Mancini | | MD | University of British Columbia | Vancouver, Canada | Site investigator | |
| Issa | Metanis | | MD | Hadassah-Hebrew University Medical Center | Jerusalem, Israel | Site investigator | |
| Ronen R. | Leker | | MD | Hadassah-Hebrew University Medical Center | Jerusalem, Israel | Site investigator | |
| Kelly | Pan | | BS | The Warren Alpert Medical School of Brown University, Brown Medical School | Providence, Rhode Island, USA | Site investigator | |
| Vishnu | Dantu | | BS | The Warren Alpert Medical School of Brown University, Brown Medical School | Providence, Rhode Island, USA | Site investigator | |
| Karl | Baumgartner | | BS | The Warren Alpert Medical School of Brown University, Brown Medical School | Providence, Rhode Island, USA | Site investigator | |
| Tina | Burton | | MD | The Warren Alpert Medical School of Brown University, Brown Medical School | Providence, Rhode Island, USA | Site investigator | |
| Regina | Von Rennenberg | | MD | Charite Universitätsmedizin-Berlin und Center for Stroke Research | Berlin, Germany | Site investigator | |
| Christian N. | Nolte | | MD | Charite Universitätsmedizin-Berlin und Center for Stroke Research | Berlin, Germany | Site investigator | |
| Richard | Choi | | MD | ChristianaCare | Newark, Delaware, USA | Site investigator | |
| Jason | MacDonald | | NP | ChristianaCare | Newark, Delaware, USA | Site investigator | |
| Reza | Bavarsad Shahripour | | MD | University of California at San Diego | San Diego, California, USA | Site investigator | |
| Xiofan | Guo | | MD | Loma Linda University | Loma Linda, California, USA | Site investigator | |
| Sebastian | Sanchez | | MD | University of Iowa | Iowa City, Iowa, USA | Site investigator | |
| Faycal | Zine-Eddine | | MD | Université de Montréal | Montreal, Canada | Site investigator | |

Supplemental Online Content: Nonauthor Collaborators
*First name, last name, and suffix (if applicable) are required and will appear in PubMed.

| *First Name and Middle Initial(s) | *Last Name | *Suffix (eg, Jr, III) | Academic Degrees | Institution | Location (city, state/province, country) | Role or Contribution, eg, chair, principal investigator | Group (if more than 1 Group listed in the byline) and/or Subgroup (eg, Steering Committee) |
|---|---|---|---|---|---|---|---|
| Maria | Fortuna Baptista | | MD | Hospital de Santa Maria | Lisbon, Portugal | Site investigator | |
| Diana | Cruz | | MD | Hospital de Santa Maria | Lisbon, Portugal | Site investigator | |
| Giovanna | De Marco | | MD | Bufalini Hospital | Cesena, Italy | Site investigator | |
| Marco | Longoni | | MD | Bufalini Hospital | Cesena, Italy | Site investigator | |
| Kim | Griffin | | MD | Mayo Clinic | Rochester, Minnesota, USA | Site investigator | |
| Lindsey | Kuohn | | MD | NYU Langone Health | New York, New York, USA | Site investigator | |
| Jennifer | Frontera | | MD | NYU Langone Health | New York, New York, USA | Site investigator | |
| Jordan | Amar | | MD | Washington University | Saint Louis, Missouri, USA | Site investigator | |
| James | Giles | | MD | Washington University | Saint Louis, Missouri, USA | Site investigator | |
| Rosario | Pascarella | | MD | Azienda Unità Sanitaria Locale-IRCCS di Reggio Emilia | Reggio Emilia, Italy | Site investigator | |
| Ilaria | Grisendi | | MD | Azienda Unità Sanitaria Locale-IRCCS di Reggio Emilia | Reggio Emilia, Italy | Site investigator | |
| Hipolito | Nzwalo | | MD PhD | Centro Hospital Universitario do Algarve | Faro, Portugal | Site investigator | |
| Amir | Molaie | | MD | University of California at Los Angeles | Los Angeles, California, USA | Site investigator | |
| Annie | Cavalier | | MD | Duke University | Durham, North Carolina, USA | Site investigator | |
| Mohammad | Anadani | | MD | Medical University of South Carolina | Charleston, South Carolina, USA | Site investigator | |
| Kimberly | Kicielinski | | MD | Medical University of South Carolina | Charleston, South Carolina, USA | Site investigator | |
| Ali | Eltatawy | | MD | University of Cincinnati Medical Center | Cincinnati, Ohio, USA | Site investigator | |
| Lina | Chervak | | MD | University of Cincinnati Medical Center | Cincinnati, Ohio, USA | Site investigator | |
| Roberto | Chulluncuy-Rivas | | MD | University of Cincinnati Medical Center | Cincinnati, Ohio, USA | Site investigator | |

Supplemental Online Content: Nonauthor Collaborators
*First name, last name, and suffix (if applicable) are required and will appear in PubMed.

| ***First Name and Middle Initial(s)** | ***Last Name** | ***Suffix (eg, Jr, III)** | Academic Degrees | Institution | Location (city, state/province, country) | Role or Contribution, eg, chair, principal investigator | Group (if more than 1 Group listed in the byline) and/or Subgroup (eg, Steering Committee) |
|---|---|---|---|---|---|---|---|
| Yasmin | Aziz | | MD | University of Cincinnati Medical Center | Cincinnati, Ohio, USA | Site investigator | |
| Ekaterina | Bakradze | | MD | University of Alabama at Birmingham | Birmingham, Alabama, USA | Site investigator | |
| Thanh Lam | Tran | | MD | University of Alabama at Birmingham | Birmingham, Alabama, USA | Site investigator | |
| Marco | Rodrigo-Gisbert | | MD | University Hospital Vall d'Hebron | Barcelona, Spain | Site investigator | |
| Manuel | Requena | | MD | University Hospital Vall d'Hebron | Barcelona, Spain | Site investigator | |
| Faddi | Saleh Velez | | MD | University of Oklahoma | Norman, Oklahoma, USA | Site investigator | |
| Jorge | Ortiz Garcia | | MD | University of Oklahoma | Norman, Oklahoma, USA | Site investigator | |
| Varsha | Mudassani | | MD | Einstein-Jefferson Healthcare Network | Philadelphia, Pennsylvania, USA | Site investigator | |
| Adam | de Havenon | | MD | Yale New Haven Hospital | New Haven, Connecticut, USA | Site investigator | |
| Venugopalan Y. | Vishnu | | MD | All India Institute of Medical Sciences | New Delhi, India | Site investigator | |
| Sridhara | Yaddanapudi | | MD | Thomas Jefferson University | Philadelphia, Pennsylvania, USA | Site investigator | |
| Latasha | Adams | | MBS | Thomas Jefferson University | Philadelphia, Pennsylvania, USA | Site investigator | |
| Abigail | Browngoehl | | MPH | Thomas Jefferson University | Philadelphia, Pennsylvania, USA | Site investigator | |
| Tamra | Ranasinghe | | MD | Wake Forest Medical Center | Winston-Salem, North Carolina, USA | Site investigator | |
| Randy | Dunston | | MD | Wake Forest Medical Center | Winston-Salem, North Carolina, USA | Site investigator | |
| Zachary | Lynch | | MD | Wake Forest Medical Center | Winston-Salem, North Carolina, USA | Site investigator | |
| James | Siegler | | MD | Cooper University | Camden, New Jersey, USA | Site investigator | |
| Silva | Mayer | | MD | Columbia University Medical Center | New York, New York, USA | Site investigator | |

Supplemental Online Content: Nonauthor Collaborators
*First name, last name, and suffix (if applicable) are required and will appear in PubMed.

| ***First Name and Middle Initial(s)** | ***Last Name** | ***Suffix (eg, Jr, III)** | Academic Degrees | Institution | Location (city, state/province, country) | Role or Contribution, eg, chair, principal investigator | Group (if more than 1 Group listed in the byline) and/or Subgroup (eg, Steering Committee) |
|---|---|---|---|---|---|---|---|
| Joshua | Willey | | MD | Columbia University Medical Center | New York, New York, USA | Site investigator | |
| Yee Kuang | Cheng | | MD | Yale New Haven Hospital | New Haven, Connecticut, USA | Site investigator | |
| Vitor | Mendes Ferreira | | MD | Hospital de Egas Moniz, Centro Hospitalar Lisboa Ocidental | Lisbon, Portugal | Site investigator | |
| Piers | Klein | | MA | Boston Medical Center | Boston, Massachusetts, USA | Site investigator | |
| Thanh N. | Nguyen | | MD | Boston Medical Center | Boston, Massachusetts, USA | Site investigator | |
| Syed Daniyal | Asad | | MD | Hartford Hospital | Hartford, Connecticut, USA | Site investigator | |
| Zoha | Sarwat | | MD | Hartford Hospital | Hartford, Connecticut, USA | Site investigator | |
| Anvesh | Balabhadra | | MD | Hartford Hospital | Hartford, Connecticut, USA | Site investigator | |
| Shivam | Patel | | MD | Hartford Hospital | Hartford, Connecticut, USA | Site investigator | |
| Thais | Secchi | | MD | Hospital Moinhos de Vento | Porto Alegre, Brazil | Site investigator | |
| Sheila | Martins | | MD | Hospital Moinhos de Vento | Porto Alegre, Brazil | Site investigator | |
| Gabriel | Mantovani | | MD | Hospital Moinhos de Vento | Porto Alegre, Brazil | Site investigator | |
| Young Dae | Kim | | MD | Yonsei University | Seoul, South Korea | Site investigator | |
| Sivani | Lingam | | MD | Kansas University Medical Center | Kansas City, Kansas, USA | Site investigator | |
| Abid | Quereshi | | MD | Kansas University Medical Center | Kansas City, Kansas, USA | Site investigator | |
| Sebastian | Fridman | | MD | Western Ontario University | London, Canada | Site investigator | |
| Alonso | Alvarado | | MD | Western Ontario University | London, Canada | Site investigator | |
| Farid | Khasiyev | | MD | Saint Louis University | Saint Louis, Missouri, USA | Site investigator | |
| Guillermo | Linares | | MD | Saint Louis University | Saint Louis, Missouri, USA | Site investigator | |
| Marina | Mannino | | MD | AOOR Villa Sofia-V. Cervello | Palermo, Italy | Site investigator | |
| Valeria | Terruso | | MD | AOOR Villa Sofia-V. Cervello | Palermo, Italy | Site investigator | |
| Sofia | Vassilopoulou | | MD | National and Kapodistrian University of Athens | Athens, Greece | Site investigator | |

Supplemental Online Content: Nonauthor Collaborators
*First name, last name, and suffix (if applicable) are required and will appear in PubMed.

| ***First Name and Middle Initial(s)** | ***Last Name** | ***Suffix (eg, Jr, III)** | Academic Degrees | Institution | Location (city, state/province, country) | Role or Contribution, eg, chair, principal investigator | Group (if more than 1 Group listed in the byline) and/or Subgroup (eg, Steering Committee) |
|---|---|---|---|---|---|---|---|
| Vasilis | Tentolouris | | MD | National and Kapodistrian University of Athens | Athens, Greece | Site investigator | |
| Manuel | Martinez-Marino | | MD | Centro Médico Nacional Siglo XXI IMSS | Mexico City, Mexico | Site investigator | |
| Victor | Carrasci Wall | | MD | Centro Médico Nacional Siglo XXI IMSS | Mexico City, Mexico | Site investigator | |
| Francisca | Indraswari | | MD | The Miriam Hospital | Providence, Rhode Island, USA | Site investigator | |
| Sleiman | El Jamal | | MD | The Miriam Hospital | Providence, Rhode Island, USA | Site investigator | |
| Shilin | Liu | | MD | University of Science and Technology | Shanghai, China | Site investigator | |
| Muhammad | Alvi | | MD | West Virginia University | Morgantown, West Virginia, USA | Site investigator | |
| Farman | Ali | | MD | West Virginia University | Morgantown, West Virginia, USA | Site investigator | |
| Mohammed | Sarvath | | MD | West Virginia University | Morgantown, West Virginia, USA | Site investigator | |
| Rami Z. | Morsi | | MD | University of Chicago | Chicago, Illinois, USA | Site investigator | |
| Tareq | Kass-Hout | | MD | University of Chicago | Chicago, Illinois, USA | Site investigator | |
| Feina | Shi | | MD | Sir Run Run Shaw Hospital of Zhejiang University Medical School | Hangzhou, China | Site investigator | |
| Jinhua | Zhang | | MD | Sir Run Run Shaw Hospital of Zhejiang University Medical School | Hangzhou, China | Site investigator | |
| Dilraj | Sokhi | | MD | Aga Khan University | Nairobi, Kenya | Site investigator | |
| Jamil | Said | | MD | Aga Khan University | Nairobi, Kenya | Site investigator | |
| Alexis N. | Simpkins | | MD PhD | Cedars Sinai Medical Center | Los Angeles, California, USA | Site investigator | |
| Roberto | Gomes | | MD | Cedars Sinai Medical Center | Los Angeles, California, USA | Site investigator | |
| Shayak | Sen | | MD | Cedars Sinai Medical Center | Los Angeles, California, USA | Site investigator | |

Supplemental Online Content: Nonauthor Collaborators
*First name, last name, and suffix (if applicable) are required and will appear in PubMed.

| ***First Name and Middle Initial(s)** | ***Last Name** | ***Suffix (eg, Jr, III)** | Academic Degrees | Institution | Location (city, state/province, country) | Role or Contribution, eg, chair, principal investigator | Group (if more than 1 Group listed in the byline) and/or Subgroup (eg, Steering Committee) |
|---|---|---|---|---|---|---|---|
| Mohammad | Ghani | | MD | University of Louisville | Louisville, Kentucky, USA | Site investigator | |
| Han | Xiao | | MS | University of California Santa Barbara | Santa Barbara, California, USA | Site investigator | |
| Narendra | Kala | | MD | The Warren Alpert Medical School of Brown University, Brown Medical School | Providence, Rhode Island, USA | Site investigator | |
| Christoph | Fretz | | MD | The Warren Alpert Medical School of Brown University, Brown Medical School | Providence, Rhode Island, USA | Site investigator | |
| Nahid | Mohammadzadeh | | MD | The Warren Alpert Medical School of Brown University, Brown Medical School | Providence, Rhode Island, USA | Site investigator | |
| Eric | Goldstein | | MD | The Warren Alpert Medical School of Brown University, Brown Medical School | Providence, Rhode Island, USA | Site investigator | |
| Karen | Furie | | MD | The Warren Alpert Medical School of Brown University, Brown Medical School | Providence, Rhode Island, USA | Site investigator | |
