# Supplementary material for: Fibromuscular Dysplasia and Spontaneous Cervical Artery Dissection
Source: JAMA Netw Open. 2025 Nov 6;8(11):e2540800. doi: 10.1001/jamanetworkopen.2025.40800 (PMC12593120; doi:10.1001/jamanetworkopen.2025.40800)
Supplement: Supplement 3. — Data Sharing Statement [file jamanetwopen-e2540800-s003.pdf]

Data Sharing Statement

Nehme. Fibromuscular Dysplasia and Spontaneous Cervical Artery Dissection. *JAMA Netw Open*. Published November 06, 2025. doi:10.1001/jamanetworkopen.2025.40800

Data
Data available: No
